# Editorial: Special Issue on the “Molecular Biology of Disease Vectors”

**DOI:** 10.3390/ijms24032881

**Published:** 2023-02-02

**Authors:** Michail Kotsyfakis

**Affiliations:** Laboratory of Genomics and Proteomics of Disease Vectors, Institute of Parasitology, Biology Centre, Czech Academy of Sciences, 37005 Ceske Budejovice, Czech Republic; kotsyfakis@paru.cas.cz; Tel.: +420-387-775-492

Arthropod disease vectors not only transmit malaria but many other serious diseases, many of which are, to a greater or lesser degree, neglected. There is therefore a need for concerted efforts to develop new means with which to prevent disease transmission. In most cases, disease transmission involves a tripartite interaction between the arthropod disease vector, the vertebrate host, and the vector-borne pathogen. This Special Issue provides a compilation of the latest research in this area, together with up-to-date information on the molecular and biochemical events that mediate this tripartite interaction.

Two papers report on the application of systems biology approaches to hard ticks, which serve as important disease vectors in the Western world. *Ixodes ricinus* ticks are distributed across Europe and are important vectors of tick-borne encephalitis as well as Lyme disease. The last decade has seen intensive efforts in characterizing and understanding the roles of long non-coding RNAs (lncRNA) in health and disease, including in vector biology [1,2]. Here, Medina et al. present an exhaustive analysis of *I. ricinus* lncRNAs based on 131 RNA-seq datasets from three different BioProjects [3]. Their data analysis suggests that lncRNAs may act as sponges (scavengers/binders) of host miRNAs and thus exert diverse biological roles related to tick–host interactions in different tick tissues. Similarly, microRNAs (miRNAs) are a class of small non-coding RNAs involved in many biological processes, including in the immune pathways that control bacterial, parasitic, and viral infections. There are little data on differentially expressed miRNAs in the black-legged tick *Ixodes scapularis* after infection with *Borrelia burgdorferi*, the causative agent of Lyme disease in the United States. Kumar et al. used small RNA sequencing and qRT-PCR analyses to identify and validate differentially expressed *I. scapularis* salivary miRNAs [4], and in doing so provided new insights into the miRNAs expressed in *I. scapularis* salivary glands in addition to paving the way for their functional manipulation to prevent or treat *B. burgdorferi* infection.

Hard ticks feed for several days or weeks on their hosts, and their saliva contains thousands of polypeptides belonging to dozens of families, as identified by salivary transcriptomic analyses [5]. Mapping coding sequences to protein databases helps to identify putative secreted proteins and their potential functions at the tick–host interface, where pathogen transmission takes place. Mans et al. analyzed the classification of tick salivary proteins given recent developments in the Alphafold2/Dali programs, and in doing so detected novel protein families and revealed new insights that connected the structures and functions of tick salivary proteins [6]. Tick saliva is a rich source of antihemostatic, anti-inflammatory, and immunomodulatory molecules that actively help ticks to finish their blood meal [7,8]. Kotál et al. presented the functional and structural characterization of Iripin-8, a salivary serpin from *I. ricinus* [9]. The first crystal structure of a tick serpin in the native state demonstrated that Iripin-8 is a tick serpin with a conserved reactive center loop that possesses antihemostatic activity that may mediate interference with a host’s innate immunity. 

Host blood protein digestion, essential for tick development and reproduction, occurs in a tick’s midgut digestive cells, driven by cathepsin proteases. Little is known about the regulation of the digestive proteolytic machinery in *I. ricinus*. In another paper from Kotál et al. the team present the functional and structural characterization of a novel cystatin-type protease inhibitor, mialostatin, from the *I. ricinus* midgut, which is likely to be involved in the regulation of gut-associated proteolytic pathways, making midgut cystatins promising targets for tick control strategies [10].

Arthropod-borne viruses, referred to collectively as arboviruses, infect millions of people worldwide each year and have the potential to cause severe disease. They are predominately transmitted to humans through the blood-feeding behavior of three main groups of biting arthropods: ticks, mosquitoes, and sandflies. The pathogens harbored by these blood-feeding arthropods are transferred to animal hosts through the deposition of virus-rich saliva into the skin. These infections sometimes become systemic and can lead to neuroinvasion as well as life-threatening viral encephalitis. Schneider et al. review the ways in which arthropod vectors influence viral pathogenesis [11]. They particularly emphasize how saliva and salivary gland extracts from the three dominant arbovirus vectors impact the trajectory of the cellular immune response to arbovirus infection in the skin. 

The increase in the global disease burden and distribution of arboviruses is driven primarily by the spread of the two key invasive disease vectors, *Aedes aegypti* and *Ae. albopictus,* and by the spread of new and re-emerging viruses through international travel. Ahmed et al. present data supporting the emergence of *Ae. albopictus* in Sudan. This is a serious public health concern and argues for urgent improvements in vector surveillance as well as control through the implementation of integrated molecular xenosurveillance [12]. The threat of major arboviral diseases in the region underlines the need for the institutionalization of the One Health strategy for the prevention and control of future pandemics.

Cysteine-rich trypsin-inhibitor-like domain (TIL)-harboring proteins are broadly distributed in nature but remain understudied in vector mosquitoes. Tikhe et al. provide new insights into the role of a TIL-domain-containing protein of the arbovirus vector *Ae. Aegypti*, called cysteine-rich venom protein 379 (CRVP379) [13]. CRVP379 was previously shown to be essential for dengue virus infection in *Ae. aegypti* mosquitoes. Here, the importance of CRVP379 is demonstrated in *Ae. aegypti* reproductive biology, which makes this molecule an interesting candidate for the development of *Ae. aegypti* population control methods. 

The PIWI-interacting RNA (piRNA) pathway, first characterized in *Drosophila,* provides an RNA interference (RNAi) mechanism with which to maintain the integrity of the germline genome by silencing transposable elements. *Ae. aegypti* mosquitoes exhibit an expanded repertoire of PIWI proteins involved in the piRNA pathway, suggesting their functional divergence. Williams et al. investigated the RNA-binding dynamics and subcellular localization of *Ae. aegypti* Piwi4 (AePiwi4), a PIWI protein involved in antiviral immunity and embryonic development [14]. Their experiments provide insights into the dynamic role played by AePiwi4 in RNAi and pave the way for future studies in order to understand PIWI interactions with diverse RNA populations.

The sole currently approved malaria vaccine targets the circumsporozoite protein that densely coats the surface of sporozoites, the parasite stage deposited into the skin of the mammalian host by infected mosquitoes; however, this vaccine only confers moderate protection against clinical disease in children, driving the search for novel candidates. Sá et al. demonstrate the importance of the membrane-associated erythrocyte binding-like protein (MAEBL) for infection by *Plasmodium* sporozoites [15]. Their data provide further insights into the role of MAEBL in sporozoite infectivity and may contribute to the design of future immune interventions.

Climate change is probably the foremost threat to human health in the 21st century. Climate directly impacts health through climatic extremes, air quality, rises in sea level, and multifaceted influences on food production systems as well as water resources. Climate also affects infectious diseases, which have played a significant role in human history—not least recently with the COVID-19 pandemic—impacting the rise and fall of civilizations in addition to facilitating the conquest of new territories [16]. Research into neglected vector-borne diseases must be a priority as the effects of climate change become ever more apparent. Together, the articles in this Special Issue highlight significant aspects of the physiology of different disease vectors, shed light on the molecular biology of a vector-borne pathogen, provide data on the tripartite interactions between vector-borne pathogens, disease vectors, and vertebrate hosts, and present evidence about the emergence of disease vectors in new geographical territories.
